# Hemorrhagic Lesions in the Central Nervous System: Toxoplasmosis in a Person Living With Human Immunodeficiency Virus Infection and Acquired Immunodeficiency Syndrome

**DOI:** 10.7759/cureus.24827

**Published:** 2022-05-08

**Authors:** Navneet Arora, Sejal Kotwani, Manika Chhabra, Mohan H

**Affiliations:** 1 Internal Medicine, Postgraduate Institute of Medical Education and Research (PGIMER), Chandigarh, IND; 2 Radiology, Postgraduate Institute of Medical Education and Research (PGIMER), Chandigarh, IND

**Keywords:** susceptibility-weighted images, cd4 cell, opportunistic infection, person living with hiv/aids (plha), toxoplasmosis

## Abstract

Central nervous system (CNS) toxoplasmosis is one of the common causes of hemorrhagic brain lesions in people living with HIV and AIDS (PLWHA), resulting in high mortality and morbidity. It has a broad clinical and neuro-radiological spectrum, which may or may not be limited to typical findings of focal and subacute neurological deficits or ring-enhancing lesions in the basal ganglia. Here, we present a case of a patient who is a newly detected person living with HIV and AIDS with a low CD_4_ cell count and classical imaging findings of central nervous system toxoplasmosis on his magnetic resonance imaging (MRI) of the brain. The incidence of opportunistic infections has been reduced after introducing highly active antiretroviral therapy (HAART); this case will be helpful to clinicians in identifying CNS toxoplasmosis as it has classical imaging findings on the MRI brain.

## Introduction

Central nervous system (CNS)-related toxoplasmosis remains one of the most common causes of high mortality and morbidity in people living with HIV/AIDS (PLWHA), with a broad spectrum of clinical and neurological manifestations. Acute toxoplasmosis is subclinical in immunocompetent individuals and shows no clinical manifestations; however, reactivation of toxoplasma causes cerebral toxoplasmosis and is characterized by focal subacute neurological defects with typical ring-enhancing brain lesions more prominently in the basal ganglia. The first case of cerebral toxoplasmosis was described between 1982 and 1983, at the beginning of the epidemic of AIDS [1]. The diminished antiparasitic T-cell response explains the reactivation of toxoplasma due to the immunosuppression caused by the human immunodeficiency virus [2]. The tachyzoites and the bradyzoites damage the walls of the small arterioles in the CNS, causing the typical findings on imaging, described in detail in the discussion, making this case important in understanding the pathophysiology of the CNS lesions in toxoplasmosis.

## Case presentation

**Figure 1:**
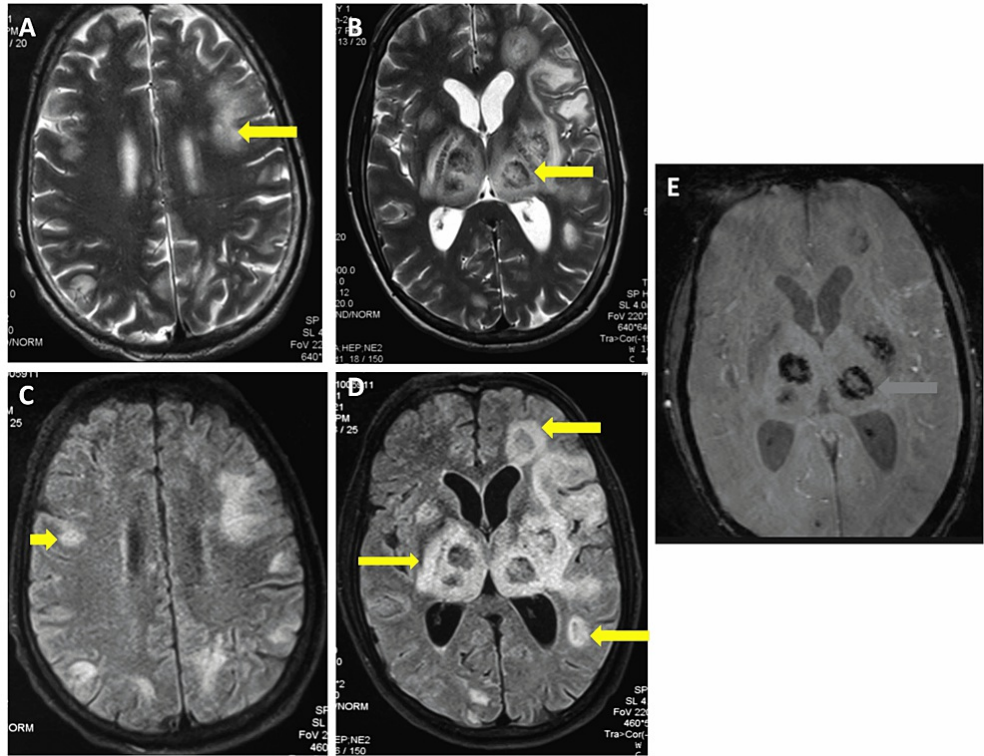
Magnetic resonance imaging of the brain (A-E) Axial T2 weighted images (A and B) and FLAIR images (C and D) show multiple hyperintense lesions (yellow arrows) in bilateral cerebral hemispheres at the corticomedullary junction, bilateral basal ganglia, and thalami, which show blooming (blue arrow) on susceptibility-weighted images (E). In an immunocompromised patient, these findings suggest toxoplasmosis.

A 42-year-old male presented to the emergency room with complaints of high-grade fever, multiple episodes of seizures, altered mental status, and bilateral upper and lower limb weakness for one week. There was no history of multiple unprotected sexual intercourse or any other high-risk behaviors. On presentation, he had a Glasgow coma score of nine. The patient was newly diagnosed with HIV/AIDS. Laboratory investigations revealed an aCD4 cell count of 37 cells/µl and a viral load of 39 copies/ml. A cerebrospinal fluid (CSF) examination revealed a total cell count of 23 (lymphocytic predominant), a glucose level of 76 mg/dl (45-80 mg/dl), and a protein content of 102 mg/dl (15-60 mg/dl). CSF Gene Xpert for *Mycobacterium tuberculosis* and India ink tests were negative; however, a nested polymerase chain reaction for *Toxoplasma gondii* was positive. Toxoplasma IgG serology was positive by ELISA. Magnetic resonance imaging (MRI) of the brain revealed multiple discrete T2/fluid-attenuated inversion recovery (FLAIR) hyperintense lesions in bilateral cerebral hemispheres at the corticomedullary junction, bilateral basal ganglia, and thalami, which showed blooming on susceptibility-weighted images (SWI) (Figure 1A-1E).

In our case, no significant contrast enhancement was seen within the lesions. An electroencephalogram was suggestive of diffuse encephalopathy changes. The patient was started on trimethoprim-sulfamethoxazole along with supportive care. He improved with the given treatment, and he was sent to the antiretroviral therapy clinic for further management.

## Discussion

*Toxoplasma gondii* is a ubiquitous protozoan parasite. It is the most common opportunistic infection in PLWHA in low and middle-income countries (LMIC), with a prevalence and burden of 89% [1]. The risk of CNS toxoplasmosis is significantly increased in patients with a CD4 count of less than 100 cells/mm^3^, seropositivity to IgG antibodies, and not receiving regular and adequate prophylaxis [3]. Presenting symptoms include headaches, mental confusion, seizures, ataxia, and visual abnormalities. MRI plays an essential role in diagnosing and differentiating this disease from other focal CNS lesions in HIV patients, such as primary CNS lymphoma, tuberculoma, and cryptococcosis. MRI shows multiple ring-enhancing lesions at the corticomedullary junction and basal ganglia with surrounding edema and blooming on SWI [4]. Intralesional blooming represents hemorrhage. Tachyzoites and bradyzoites lodging within necrotizing abscesses in toxoplasmosis may damage the walls of small arterioles and cause capillary thrombosis or immune-mediated vasculitis, leading to intralesional hemorrhage which can be easily identified as blooming on SWI [5,6]. As seen in our case, multiple rounded hemorrhages associated with perilesional edema can be a unique radiological finding. Blooming on SWI can help differentiate toxoplasmosis from CNS lymphoma, as typically untreated lymphoma lesions do not show hemorrhage [7,8]. Diagnosis can be confirmed by CSF PCR, which has 60% sensitivity and 100% specificity [9]. However, toxoplasmic encephalitis can be presumptively diagnosed based on clinical presentation, low CD4 count, toxoplasma IgG seropositive (secondary reactivation), and radiological appearance. Other diseases that can mimic cerebral toxoplasmosis may be Nocardia species, Varicella-Zoster, Aspergillus species, CNS tuberculosis, and primary CNS lymphomas (PCNSL). Brain biopsy is reserved for cases in which alternative diagnoses cannot be excluded or there is no response after 10-14 days of antiparasitic therapy. The first-line treatment includes pyrimethamine-sulfadiazine; however, trimethoprim-sulfamethoxazole is given due to unavailability in low and middle-income countries, which is equally effective.

## Conclusions

Though the introduction of highly active antiretroviral therapy (HAART) has markedly decreased the incidence of cerebral toxoplasmosis, there is still a considerable disease burden present in LMIC. Reactivation of toxoplasma, rather than primary infection, is the culprit causing the symptomatic disease, which leads to considerable mortality and morbidity. MRI plays an essential role in diagnosing this disease and shows multiple ring-enhancing lesions with surrounding edema and blooming on susceptibility-weighted imaging. Multiple rounded hemorrhages (peripheral) associated with perilesional edema are a unique radiological finding seen in the MRI. Trimethoprim-sulfamethoxazole is used for primary prophylaxis, initial therapy, and secondary prophylaxis of HIV-related toxoplasmosis.
